# Sleep-Related Problems in Night Shift Nurses: Towards an Individualized Interventional Practice

**DOI:** 10.3389/fnhum.2021.644570

**Published:** 2021-03-16

**Authors:** Valentina Alfonsi, Serena Scarpelli, Maurizio Gorgoni, Mariella Pazzaglia, Anna Maria Giannini, Luigi De Gennaro

**Affiliations:** ^1^Body and Action Lab, IRCCS Fondazione Santa Lucia, Rome, Italy; ^2^Department of Psychology, Sapienza University of Rome, Rome, Italy

**Keywords:** night shift work, nurses, sleep problem, sleepiness, health, fatigue, shift scheduling

## Abstract

Rotating shifts (mostly 8- or 12-h) are common among nurses to ensure continuity of care. This scheduling system encompasses several adverse health and performance consequences. One of the most injurious effects of night-time shift work is the deterioration of sleep patterns due to both circadian rhythm disruption and increased sleep homeostatic pressure. Sleep problems lead to secondary effects on other aspects of wellbeing and cognitive functioning, increasing the risk of errors and workplace accidents. A wide range of interventions has been proposed to improve the sleep quality of nurses and promote an increase in attention levels. In recent years, particular attention has been paid to individual and environmental factors mediating the subjective ability to cope with sleep deprivation during the night shift. Given the predictive role of these factors on the negative impact of a night shift, an individualized intervention could represent an effective countermeasure by ensuring suitable management of shift schedules. Therefore, the aims of this mini-review are to: (a) provide an updated overview of the literature on sleep problems in night shift nurses and their adverse consequences; and (b) critically analyze the psychosocial factors that mediate the negative impact of shift work with the ultimate goal of defining an effective countermeasure based on an individualized approach.

## Introduction

Nowadays, nearly a fifth of the global workforce is engaged in shift work (40% in Europe; Ferri et al., 2016; Parent-Thirion et al., 2016). In the healthcare sector, working in shifts ensures the continuity of patient care around the clock. There is mounting evidence that night shift work has a significant impact on health and performance in medical personnel due to the alteration of natural homeostatic and circadian sleep processes (Boivin and Boudreau, 2014; Sagherian et al., 2017; Ganesan et al., 2019), which can seriously compromise public safety of both patients and medical staff by increasing the risk of errors and workplace accidents (Di Muzio et al., 2019; Larsen et al., 2020; Smith et al., 2020).

The International Classification of Sleep Disorders (American Academy of Sleep Medicine, 2005) estimates that the syndrome known as “shift work disorder” is experienced by 20–30% of shift workers (Drake et al., 2004; Flo et al., 2012). The most common shift schedule is organized in a continuous rotating fashion, that is, morning, afternoon, and night shifts (Lin et al., 2014). Regardless of the schedule, a complex interaction between internal and external factors explains the adverse consequences on individual wellbeing (Booker et al., 2018).

This review focuses on the most widespread and studied population in the context of shift work: nursing staff (Ball et al., 2015). First, we critically discuss the recent evidence surrounding the effects of night shift work on nurses, focusing on sleep-related problems and consequences on both individual and institutional levels. Then, we briefly describe the current countermeasures with the ultimate goal of filling the gap between the growing knowledge about the key role of inter-individual variability in response to shift work and the development of effective tailored interventions.

## Sleep-Related Effects of Night Shift Work in Nurses

Internal (“biological”) and external (“zeitgebers”) circadian clocks (Silver and Schwartz, 2005) are synchronized to allow us optimal performance during the day and restorative sleep at night (Achermann et al., 2017). In night-shift workers, sleep displacement leads to the so-called “circadian misalignment” (Boivin and Boudreau, 2014). This phenomenon refers to the lack of entrainment between internal bodily rhythms and the night schedule (Czeisler and Buxton, 2010). In turn, the circadian misalignment alters natural sleep homeostasis. Daytime sleep duration is significantly shortened (Geiger-Brown et al., 2012; Kaliyaperumal et al., 2017), and typical insomnia symptoms are commonly reported in this population (Shao et al., 2010). The alteration of circadian and homeostatic processes not only directly affects sleep but also causes several medical and non-medical problems.

Chronic partial sleep deprivation represents an important risk factor for developing various diseases among nurses (for review, see Rosa et al., 2019), primarily cardiovascular diseases (Yu et al., 2016), type 2 diabetes (Hansen et al., 2016), metabolic syndrome (Kecklund and Axelsson, 2016), gastrointestinal disorders (Lu et al., 2006), and cancer (Hansen and Stevens, 2012). Additionally, the interference with regular meal routines and the reduced physical activity of working the night shift contributes to worsening these pathological conditions (Thompson et al., 2017). Mental health may also be affected by the persistent stimulation of the hypothalamic-pituitary–adrenal axis due to frequent exposure to external stressors in night workers, leading to high stress-response reactivity (Kalmbach et al., 2015). In the long-term, night shift work may increase the risk for mental disorders, especially depression and anxiety (Mealer et al., 2012).

At the same time, studies using different methodology confirm the adverse outcomes on behavioral and cognitive performance (Zion and Shochat, 2018; Behrens et al., 2019; Ganesan et al., 2019). Indeed, nurses often complain of sleepiness and fatigue symptoms during night shifts (Wilson et al., 2019), as the biological circadian clock promotes sleep during that period. This condition naturally leads to a greater likelihood of errors and accidents (Di Muzio et al., 2019; James et al., 2020), which is estimated at 30% higher compared to morning shift workers (de Cordova et al., 2016).

Given the complex interaction between individual and environmental factors in determining the overall effects of shift work in nurses, caution should be used in generalizing these results. Indeed, some studies reported no significant changes in sleep quality or health (Costa, 2003; Fietze et al., 2009), as a function of the specific population or working condition considered in the investigation.

## Current Interventions for Night Shift Workers

Several studies show an improvement in vigilance following the exposure of workers to bright light (Huang et al., 2013; Jensen et al., 2016) and pave the way for combined light-base intervention to improve performance and reduce fatigue (Olson et al., 2020). Recently, novel strategies based on blue-enriched light in the workspace have received increasing interest (Sletten et al., 2017; Sunde et al., 2020). Otherwise, wearing sleep goggles appears to effectively prevent vigilance increase during the daytime recovery period (Eastman et al., 1994).

Another countermeasure closely related to circadian misalignment is the administration of exogenous melatonin in the morning to promote daytime recovery sleep (Sharkey et al., 2001). This exerts an antithetical effect to that of light, advancing circadian rhythms (Yoon and Song, 2002). However, although the phase-shifting properties of exogenous melatonin are well-known, its soporific effect is still debated (Smith and Eastman, 2012).

Concerning the increased homeostatic sleep drive during night work, it has been observed that planning strategic naps before or during the night shift may counteract the rise of sleep pressure and reduce perceived sleepiness (Lovato et al., 2009). However, this represents a controversial issue, especially concerning the definition of strategic nap length and timing. A brief prophylactic nap of 20–30 min is strongly recommended to prevent the impact of subsequent sleep loss (Geiger-Brown et al., 2012; Oriyama et al., 2013; Tempesta et al., 2013). Differently, longer napping during the night shift could be dangerous because of sleep inertia, greatest at night (Scheer et al., 2008).

The use of psychostimulants during the night shift, such as caffeine or medications, immediately attenuates nocturnal sleepiness (Schweitzer et al., 2006; Czeisler et al., 2009), but presents some remarkable drawbacks. Indeed, psychostimulants lead to a significant deterioration of the following daytime sleep, which is essential for adequate recovery (Carrier et al., 2007). An alternative option is to directly act on daytime sleep through the administration of hypnotic drugs (Schweitzer et al., 1991; Walsh et al., 1995). However, taking sedatives does not address the decline of alertness during night work, so it is only a partial solution.

Acting on shift scheduling to reduce the negative impact of night work constitutes a long-standing issue. Several factors need to be considered to identify “optimal” schedule planning, and many options have been proposed over the years using mathematical or heuristic models (Ernst et al., 2004).

Permanent shift schedules have been argued to mitigate the adverse effects on sleep, maximizing the circadian clock’s adjustment (Wilkinson, 1992). On the other hand, fixed schedules negatively impact the night shift worker’s social life (Folkard, 2008) and can lead to burnout (Shahriari et al., 2014). Typical shift systems involve 2- and 3-shift rotations, having 12- and 8-h shifts, respectively (Lin et al., 2014). Rotating 12-h shifts (day-night) is associated with fewer sleep disturbances in nurses than 8-h shifts (Costa et al., 2014). However, longer working hours may increase fatigue symptoms and the need for adequate rest (McDowall et al., 2017). The increase of absence due to sickness is also related to working shifts of 12 h or more (Dall’Ora et al., 2019). Besides, the clockwise direction of shifts (forward rotation) is considered more acceptable by nigh-time workers than the counterclockwise direction (Wilson, 2002).

In the last decade, novel intervention types have been conducted to improve sleep quality in nurses, for example, aroma-inhalation therapy, physical activity interventions, stress-management programs, and cognitive-behavioral therapy (for review, see Kang et al., 2020), although the beneficial effects were small.

## Future Directions in The Management of Night Shift Nurses

**Figure 1:**
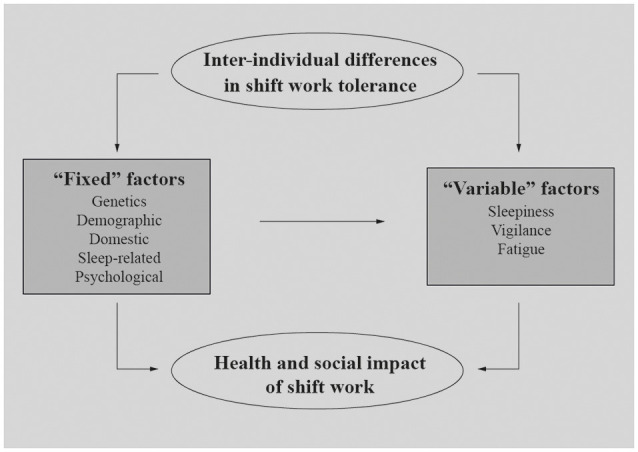
“Fixed” and “Variable” inter-individual factors potentially explaining health and social impact of night shift work on nurses.

In recent years, several studies have highlighted the existence of inter-individual differences in shift work response (Booker et al., 2018; Zion et al., 2018). Indeed, the effects on sleep are not universal, but they are mediated by some factors determining overall resilience and vulnerability. We can divide these inter-individual factors into two main groups (Figure 1): (1) “fixed” factors related to stable genetic, demographic, and psychosocial characteristics and (2) “variable” factors related to state-dependent conditions.

### “Fixed” Individual Factors

Genetics research revealed specific genetic components indirectly related to the individual resistance to the adverse effects of shift work (Sookoian et al., 2007; Viola et al., 2007; Reinberg and Ashkenazi, 2008). However, more direct investigations on the relationship between genetics and shift work tolerance are needed and strongly encouraged to broaden the individual difference perspective (Saksvik et al., 2011).

The positive relation between aging and the adverse effects of shift work has been extensively demonstrated (Costa and Sartori, 2007). Some biological aspects could explain this relation, such as the increased tendency toward “morningness” in elderly people (Härmä and Kandolin, 2001), difficulties in circadian adjustment (Zeitzer et al., 2007), and poor sleep quality affecting this population (Gander and Signal, 2008). On the other hand, years of experience in working night shifts seem to represent a protective factor (Bonnefond et al., 2006), probably thanks to improved coping strategies over time (Øyane et al., 2013).

Research on gender difference shows that women shift workers are more affected by the adverse effects of night shift (Han et al., 2016), but the findings are inconsistent across studies (Admi et al., 2008; Flo et al., 2012).

Domestic factors interfering with shift work focus on family-related responsibilities, such as taking care of children or elderly parents. The presence of children living at home positively correlates with sleep disruption and impatience among night workers (Korompeli et al., 2013), especially in women. Even taking care of elderly family members is a strong predictor of an increased need for recovery from work (Rotenberg et al., 2011).

Given the crucial impact of shift work on sleep, protective aspects such as good sleep quality and the natural tendency to be more active in the evening play a key role in mediating adverse outcomes (Åkerstedt et al., 2014). Reduced sleep quantity and quality seem to predict greater subjective sleepiness (Geiger-Brown et al., 2012). A recent study by Di Muzio et al. (2020) showed the critical role of sleep quality (as assessed by the Pittsburgh Sleep Quality Index, PSQI; Curcio et al., 2013) in exacerbating the decline of psychomotor vigilance in a sample of night shift nurses. Also, individual chronotype affects night shift tolerance, although the exact nature of this relationship is still a matter of debate (Saksvik et al., 2011). Also, findings of a recent study by Zion and colleagues identified pre-sleep arousal as a sensitive marker of adaptation to the night shift (Zion et al., 2018).

Certain personality traits are coupled with better night shift tolerance, such as high hardiness (Flo et al., 2012) and low neuroticism (Costa et al., 2014). These factors may indicate the subjective ability to prevent burnout and withstand working hours outside a typical schedule. Furthermore, a wide range of coping responses (e.g., health practice, social support, cognitive strategies) is positively associated with nurses’ management of job stressors (Tahghighi et al., 2017). Conversely, unhealthy coping strategies, such as alcohol and other substances, are common among shift workers (Schluter et al., 2012).

Keeping all these inter-individual differences in mind could allow us to identify stable predictors of the negative impact of shift work (genetic predisposition, older age, female gender, less experience with shift work, domestic duties, poor sleep quality, neuroticism, and unhealthy coping strategies).

In addition to the above-mentioned factors, inter-individual differences in vulnerability to the adverse effects of sleep deprivation were also reported, such as cognitive deficits (Van Dongen, 2006), neuroendocrine, immune, and oxidative stress (Faraut et al., 2013), the cancer-promoting mechanism (Haus and Smolensky, 2013). Moreover, the flexibility of sleep habits was found to be predictive of subjective quality of daytime recovery sleep in other shift workers population (e.g., novice police officers; Lammers-van der Holst et al., 2016).

Taken together, all these factors contribute to define the individual worker’s ability to manage the side effects of shift work and determine the efficacy of the adopted coping strategies (Savic et al., 2019). First, a timely estimation of the individual workers’ tolerance may help allocate workers to a certain shift pattern. From a practical viewpoint, we should consider the protective and risk factors to create a strategic shift scheduling based on person-specific characteristics, rather than random assignment. Specifically, an accurate initial assessment through interviews and valid instruments (e.g., PSQI) may help identify resilient shift workers who are more suited to managing non-traditional shifts. Some innovative schedule arrangements based on a participative approach have already been implemented in health organizations (Barrett and Holme, 2018; Karhula et al., 2019). However, evidence-based studies on long-term efficacy on health and ergonomics are still missing (Slanger et al., 2016). For this purpose, in the recent Working Time Society consensus statements (Ritonja et al., 2019) have been outlined the most relevant factors of the individual adjustment to shift work and the most important area for future research (Ritonja et al., 2019).

Second, identifying the individuals most prone to developing sleep problems could allow managers to select a subgroup of workers to be assigned to specific prevention and treatment programs. For instance, sleep hygiene interventions adapted to the atypical sleep condition could be implemented (Shriane et al., 2020), especially among the most at-risk categories, such as newly graduated nurses (Epstein et al., 2020).

Last, the early recognition of nurses having good resilience would be helpful not only to reduce the incidence of physical and mental problems in this population but also to improve the quality of patient care and levels of job satisfaction (Ohayon et al., 2002).

### “Variable” Individual Factors

Another group of individual factors mediating nurses’ wellness and performance refers to sleepiness, vigilance, and fatigue levels during the night shift. Such aspects do not represent trait-like factors but are strictly dependent on specific working time and context. These factors may interfere with the correct execution of work demands, frequently leading to occupational accidents and medication errors (Di Muzio et al., 2019).

Sleepiness is one of the most common complaints of night shift nurses (Sallinen and Kecklund, 2010), especially toward the end of the shift (Åkerstedt et al., 2014). Given the predictive role of subjective sleepiness in the occurrence of medical errors (Surani et al., 2015), it should be an indicator frequently monitored. There are many available tools to easily assess subjective sleepiness at different time points, such as Karolinska Sleepiness Scale (KSS; Åkerstedt and Gillberg, 1990) or the Stanford Sleepiness Scale (SSS; Hoddes et al., 1972). However, such subjective instruments are not intended to provide a reliable risk cut-off, but rather to increase the self-awareness of the time-of-day sleepiness pattern (Åkerstedt et al., 2014).

As expected, excessive sleepiness during night shift hours is associated with decreased vigilance levels and alertness (Surani et al., 2015; Behrens et al., 2019). Consequently, decisional and attentional processes undergo an inevitable deterioration (Tempesta et al., 2013; Scott et al., 2014). Also, in this case, monitoring can be crucial to prevent workplace accidents. In this regard, inexpensive and free software such as the PC-Psychomotor Vigilance Task (Reifman et al., 2018) could provide real-time individualized predictions of vigilance levels.

Another common complaint of night shift nurses is fatigue, a symptom closely related to sleepiness and consisting of feeling tired and without energy (Smith-Miller et al., 2014). Both acute and chronic fatigue in nurses may result in physical and cognitive decline (Smith-Miller et al., 2014). Persistent poor sleep quality may lead to increased fatigue, which in turn can negatively impact patient and staff safety (Sagherian et al., 2017). Fatigue symptoms can be easily monitored using specific questionnaires (e.g., Chalder Fatigue Scale, CFQ; Jackson, 2015).

Hence, the healthcare system should monitor each variable to prevent subjective suffering and, mostly, workplace accidents. For this to be achieved, workers and managers should be properly informed and actively involved. First, nurses should be rapidly trained to recognize the behavioral manifestation of excessive daytime sleepiness and know its potential consequences. Moreover, employers may adopt a systematic method to monitor employees’ work-related conditions and to establish a culture of prevention; a suitable tool such as wrist actigraphy (Smith et al., 2018) could be useful in objectively monitoring specific parameters (e.g., sleep fragmentation index), possibly affecting daytime sleepiness in addition to other factors (Stepanski, 2002).

Recently, bio-mathematical models yielding objective measures of fatigue risk in nurses have been implemented (Sagherian et al., 2018). However, this method remains rarely used in the healthcare setting compared to other sectors (e.g., aviation and transportation).

In short, recognizing and controlling the risk of alertness decline reduces the likelihood of preventable injuries and accidents, thanks to the possible adoption of prompt and effective countermeasures (e.g., prophylactic naps).

## Conclusions

Since shift work is considered inevitable to ensure continuity of care in hospital settings, and nurses represent the largest healthcare workforce (Ball et al., 2015), it is essential to create a low-risk environment. The COVID-19 pandemic has exacerbated the already known critical situation of a healthcare system overwhelmed by unexpected emergencies (e.g., Cao et al., 2020).

Certainly, the achievement of ideal night shift management represents a significant challenge for healthcare organizations. However, the entire community would benefit, from the single employees or patients to the whole healthcare system.

To sum up the raised issues in a future research agenda, we emphasize the following points:

– Deeper investigation on trait-like factors distinguishing “high” and “low” shift work tolerance, especially genetic factors;
– Implementation of bio-mathematical models yielding objective measures of vigilance and sleepiness during the night shift;
– Effectiveness studies to identify optimal strategies promoting the individual adaptation to night shift work;
– Evidence-based studies to assess the feasibility of the proposed solutions in the operational environment.

In conclusion, we suggest that the modulatory factors discussed above could pave the way for an individualized approach in managing night shift nurses from a perspective of both prevention and early intervention.

## Author Contributions

LDG and VA made substantial contributions to the conception and design of the work. LDG, VA, SS, MG, MP, and AG contributed by drafting the work and revising it critically for important intellectual content, responsible for the final approval of the version to be published, and agreed to be accountable for all aspects of the work in ensuring that questions related to the accuracy or integrity of any part of the work are appropriately investigated and resolved. All authors contributed to the article and approved the submitted version.

## Conflict of Interest

The authors declare that the research was conducted in the absence of any commercial or financial relationships that could be construed as a potential conflict of interest.
